# Filamentous multiscale characterization of the cultivation of *Actinomadura namibiensis* pellets for model-based prediction of labyrinthopeptin A1 biosynthesis

**DOI:** 10.1186/s12934-026-03088-6

**Published:** 2026-08-11

**Authors:** Qiyue Liu, Zuzanna Justyna Kozanecka, Jintian Liu, Katrin Dohnt, Rainer Krull, Markus Böl

**Affiliations:** 1 Institute of Mechanics and Adaptronics, Technische Universität Braunschweig, 38106 Braunschweig, Germany; 2 Institute of Biochemical Engineering, Technische Universität Braunschweig, 38106 Braunschweig, Germany; 3 Center of Pharmaceutical Engineering, Technische Universität Braunschweig, 38106 Braunschweig, Germany

**Keywords:** *Actinomadura namibiensis*, Multiscale experiments, Shake flask culture, Filamentous microorganisms, Dissolved oxygen tension, Labyrinthopeptin A1, Microbial modeling

## Abstract

The filamentous actinomycete *Actinomadura namibiensis* has attracted increasing interest as the only known natural producer of the carbocyclic lantibiotic labyrinthopeptin A1, a secondary metabolite with a broad antiviral spectrum of activity. Developing a model of microbial growth and product formation at the pellet level requires a deeper mechanistic understanding of the interactions between cultivation conditions, substrate availability, and oxygen limitation. These factors influence the cultivation process and product formation. As part of this work, a predictive model in the form of diffusion–reaction equations is developed to quantitatively describe nutrient uptake, oxygen diffusion limitation, and pellet growth. This framework couples the consumption kinetics of carbon sources, such as glucose and glycerol, to pellet growth, maintenance metabolism, and the formation of the secondary metabolite labyrinthopeptin A1. The model is calibrated based on the results of multiscale experiments, i.e., offline analysis of shake flask cultures and oxygen profiling of pellets at different time points under various conditions. This forms the basis for understanding nutrient uptake, metabolic heterogeneity, and mass transfer limitations within the pellets. Thus, the model provides a quantitative basis for predicting productivity. The validated multiscale model reproduces the experimental results well and can be used to predict pellet growth, substrate uptake, and product formation.

## Introduction

Filamentous microorganisms are important natural producers of valuable compounds, including bulk chemicals, enzymes, and pharmaceutical ingredients. Because of their industrial and medical importance, optimizing the cultivation of these organisms has long been a major research focus [1–5]. Their micromorphology and macromorphology are highly complex and depend strongly on the strain and product. This has led to extensive studies with model organisms, such as *Aspergillus* [6–8]. Additionally, filamentous bacteria are a rich source of secondary metabolites, such as penicillin, mevinolin, and fumagillin [5, 9]. These metabolites are promising candidates for new antimicrobial and antiviral agents, especially in light of increasing antibiotic resistance [10–14].

One example is labyrinthopeptin A1, a lantibiotic produced by the bacterium *Actinomadura namibiensis* that exhibits broad-spectrum antiviral activity against HIV, HSV, Zika, and dengue viruses [15–18]. *A. namibiensis* was cultivated in both shake flasks and 10 L stirred tank bioreactors [19], with maximum product titers of 325 mg/L observed in shake flasks [13]. Labyrinthopeptin A1 is a second metabolite whose production is initiated only after the transition from the growth phase to the production phase. In this cultivation system, this transition typically occurs after approximately three days of cultivation, following glucose depletion and the onset of glycerol consumption [20]. The process is time-consuming to optimize due to the late onset of secondary metabolism and the complex mechanisms involved during cultivation. These mechanisms are mainly governed by four coupled factors: Substrate supply, fluid dynamics, pellet morphology, and mechanical contacts [20, 21]. With respect to pellet morphology, the growth-related structural changes are determined by substrate availability. Furthermore, hydrodynamic conditions can significantly alter pellet shape by causing collisions between pellets or between pellets and the shake flask walls. Consequently, exhaustive sampling of the process parameters is neither feasible nor efficient for capturing these coupled interactions and the resulting process dynamics. Therefore, predictive modeling of key pellet kinetics of *A. namibiensis*, such as biomass growth and product formation, is essential to accelerate process optimization and improve product titers.

Current models of filamentous growth can be divided into three categories based on scale: Pellet scale, lab scale and pilot scale [22]. At the pellet scale, the temporal evolution of pellet morphology (e.g., diameter and hyphae fraction) is usually described by an evolution equation linking structural changes to nutrient availability and spatial constraints [23–28]. Although these models capture radial heterogeneity within pellets, they generally lack experimental validation [23–26, 28]. At the lab scale, modeling efforts focus on mass transfer from the culture medium into the pellet [24, 28, 29]. Using volume averaging and homogenization theory, the hyphal network is treated as a porous medium where substrate transport is described by Fick’s law in diffusion–reaction equations or Navier–Stokes equations. The complexity of these models depends on the components considered, such as intracellular or extracellular fluid [30], oxygen [31], or biomass formation [24]. At the pilot scale, the emphasis shifts toward the statistical behavior of pellet populations. For instance, Tough et al. [32] described the changing pellet size distribution over time using population balance equations. This statistical dynamical approach typically represents the evolution of pellet size and number by introducing birth and death terms [33]. This approach can be extended further to capture more detailed morphological transitions by correlating formation rates with additional defined morphological parameters [34]. Such extended models have been calibrated using measured biomass, substrate, and product concentrations [27].

However, there is still a gap between these three levels. Models at pilot scale lack the intrinsic hyphae-associated mechanisms inside the pellet, models at lab scale neglect the supply of substrate from the environment, and microscopic models at pellet scale are unsuitable for experimental validation. They cannot directly explain the effects observed at the lab level, such as the nutrition uptake, biomass development, oxygen consumption, etc. In order to bridge the gap between these levels and explain the uninterpreted lab level, it is necessary to use models at pellet scale to integrate knowledge at the hyphae level. This modeling aims to describe mass transport and growth within individual pellets. Substrate diffusion limits biomass activity in these models [31, 35–39]. These models describe the radial distribution of oxygen concentration and active biomass in relation to the effective diffusivity within the hyphal network. They also consider the impact on pellet size and internal morphology. Important parameters, such as effective diffusivities and oxygen uptake rates, are typically estimated from pellet scale experiments (e.g., microelectrode profiling measurements) or derived from structural data obtained through image analysis and X-ray microtomography.

Unlike models at pilot scale, which capture population fluctuations, and lab scale models, which focus on hyphal development, pellet scale models use an averaged approach to describe the overall process based on the behavior of one representative pellet. The pellet is geometrically simplified to a sphere, and the inhomogeneous hyphal network inside is represented by the porosity of hyphal fraction parameters. Thus, the structure-dependent behavior of pellets can be described by a one-dimensional diffusion–reaction equation that links internal mass transport and biochemical activity [35–38]. However, many studies only consider the hyphal domain and neglect the surrounding fluid phase [23, 28, 31]. They typically impose a simple Dirichlet boundary condition at the pellet surface. This assumption fixes the substrate concentration at the pellet boundary, ignoring dynamic changes in the surrounding medium. These changes include oxygen uptake by microbial consumption and replenishment by aeration. However, in real cultivation systems, the substrate concentration decreases over time, and oxygen is consumed by the biomass and continuously supplied through gas-liquid mass transfer [20, 29, 31, 40].

The number and type of variables included in a filamentous pellet model determine its complexity, as each kinetic or transport process introduces additional fields that must be solved. Most approaches use a mathematical formulation consisting of a diffusion term that represents biological reactions. The diffusion term is generally based on Fick’s law and assumes radial mass transport within the spherical pellet [35–37]. Recent studies by Schmideder et al. [39] and Deffur et al. [31] have addressed the internal morphological heterogeneity of individual pellets by linking local diffusion coefficients to the measured hyphal density or porosity from *μ *-CT data. The reaction terms describe the most important biological mechanisms occurring inside the pellet. Growth is usually formulated using Monod kinetics, whereby the specific growth rate depends on limiting substrates, typically oxygen or a carbon source [35, 36, 38]. Substrate consumption is directly coupled to growth via a first-order or Monod-type sink term. Further consumption due to respiration and maintenance is accounted for by additional oxygen sink terms [37, 38]. Cell degradation or death is usually modeled using a first-order decay rate that depends on the local substrate concentration. This allows for the prediction of inactive or lysed regions in the pellet core [27, 29]. In most models, biomass formation is the modeled product, while the synthesis of primary and secondary metabolites is not explicitly considered. Some morphologically structured population models have linked pellet morphology to overall metabolite production at the reactor scale [26, 29], but spatially resolved descriptions of metabolite formation within individual pellets are still lacking.

The aim of this study is to develop a microbial model that captures the dynamics of pellet growth, substrate consumption, and product formation in filamentous microorganisms. Unlike existing approaches, which rely on biomass-based formulations or focus solely on oxygen uptake, the proposed model integrates internal oxidation processes and links the consumption of the carbon sources glucose and glycerol due to the diauxic shift. The model distinguishes between growth- and maintenance-related metabolism, thus reflecting more realistic metabolic requirements during pellet development. Furthermore, the proposed calibration approach takes into account the changing physiological state of the pellet during cultivation by introducing an internal variable. Ultimately, this framework provides a basis for understanding the metabolic dynamics of pellets and predicting the concentration of the secondary metabolite labyrinthopeptin A1.

## Materials and methods

### Cultivation and offline analysis

All experiments were conducted using the *A. namibiensis* strain (DSM 44197). The cultivation procedure and conditions, the composition of the culture media, and the preparation of cryocultures are described in Kozanecka et al. [20]. In contrast to the procedures there, the first pre-culture was homogenized under the same conditions as the second pre-culture.

**Fig. 1:**
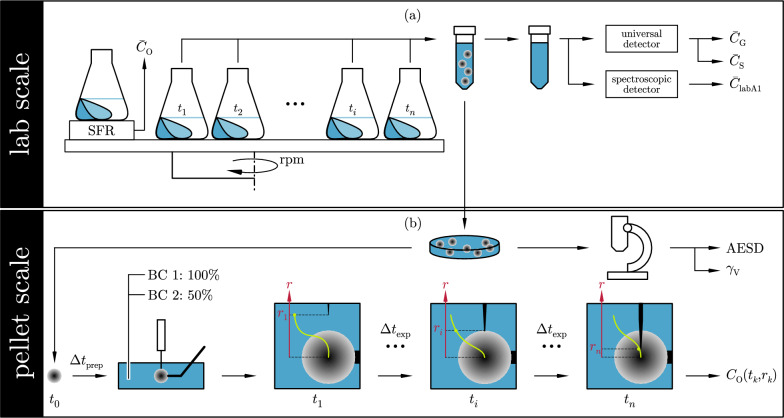
Flow diagram of the multiscale experiments: **a** Online measurement of DOT and offline analysis of the concentrations of substrates and products at the lab scale, and **b** cell counting, morphology analysis, and oxygen profiling at pellet scale. *Abbreviations:* SFR: shake flask reactor, $\bar{C}_\textrm{G}$: glycerol concentration, $\bar{C}_\textrm{S}$: glucose concentration, $\bar{C}_\textrm{labA1}$: labyrinthopeptin A1 concentration, BC 1/2: Boundary condition featuring 100%/50% bulk concentrations of oxygen, AESD: Area-equivalent spherical diameter, $\gamma _V$: relative volume ratio

The process flow for the experimental investigations on different scales (lab and pellet scale) is shown in Fig. 1.

Glucose and glycerol concentrations as well as labyrinthopeptin A1 titers, were analyzed by high-performance liquid chromatography under the measurement conditions [20]. The analysis of cell dry weight concentration was performed according to the recent protocol [20]. Dissolved oxygen tension (DOT) was monitored online using optical oxygen sensors integrated into the shake flasks (SP-PSt3-NAU and Shake Flask Reader, PreSens Precision Sensing GmbH, Germany).

### Oxygen microprofiling measurements

The experimental setup detailed described in Kozanecka et al. [20] was used for oxygen microprofiling measurements across the radius of *A. namibiensis* pellets. For the present study, the setup was expanded to include two manually adjustable gas flow controllers (GCA-C9SA-FA25 red-y, Voegtlin Instruments GmbH, Bad Koetzting, Germany) for adjusting the air-nitrogen gas mixture. This modification allowed for variation in the boundary conditions around the pellet, namely 50% and 100%bulk concentration of oxygen. Prior to the measurements, a pellet was selected under a digital microscope (EVOS xL AMG, Bothell, USA). After placing the immobilized pellet in a flow cell filled with a cultivation medium, a waiting/preparation time of $\Delta t_\mathrm{{prep}}=10$ min was realized before starting the automatic profiling. The measurements were performed with the following parameters: Step size 10 *μ *m and time increment of $\Delta t_\mathrm{{exp}} =15$ s, taking into account the 5-second waiting time before each measurement and the 10-second measurement duration. The flow was set to a Reynolds number of *Re = 127~± ~11*. In addition, the pellet edge was visually estimated while the measurement was tracked with an industrial camera (RS-OV7949-1818, Conrad Electronics SE, Hirschau, Germany).

The oxygen profiles were analyzed to determine the position of the boundary and center of the pellet. The edge of the pellet was identified as the point with the steepest negative slope in the profile. This position was then compared with the visual observations recorded during the measurement to ensure consistency. Using the area-equivalent spherical diameter (AESD) obtained from microscopic image analysis, the center of the pellet was calculated from the determined radius. The oxygen concentration at this point was verified to ensure that it corresponded to the lowest value in the profile. If this was not the case, the position of the pellet boundary determined according to the profile slope was manually adjusted to obtain a physically consistent profile shape.

### Cell counting

During cultivation, the total number of pellets was determined by volumetric extrapolation. A 1 mL aliquot was taken from 100 mL of homogenized culture medium and diluted with 0.9% (w/v) sodium chloride solution using dilution factors of 14, 25, or 27, depending on the pellet concentration. For pellet counting using digital microscopy and subsequent image analysis, a subsample of the diluted solution (200-400 *μ *L, depending on the pellet concentration) was scanned using a digital microscope with a motorized stage (Smartzoom 5, Zeiss, Oberkochen, Germany). The resulting object count in the subsample was then used to estimate the total pellet population based on the applied dilution factor. This sampling was performed at five different time points (0/24/48/120/144 h) in two independent runs.

## Kinetic modeling of filamentous pellets

The model presented here considers *A. namibiensis* pellets in a spherical domain as a continuous distribution of hyphal fractions. The interaction of the pellets with their environment arises through the transport of substrates and oxygen through the porous pellet structure. Therefore, the model domain considers both the pellet and the surrounding fluid. For the pellet growth kinetics considered here, glucose, glycerol, and oxygen are the dominant limiting substrates. The corresponding uptake rates follow the stoichiometry of chemical oxidation and reflect the experimentally observed diauxic shift, in which glucose is consumed preferentially before glycerol.

### Design of the model domain

The model proposed in this study aims to explain the relationship between productivity and substrate consumption by disregarding minor changes in pellet dimensions and their apparent variance during cultivation. Therefore, a homogenized approach was chosen to model the associated processes. This approach does not take into account the morphological variance and temporal evolution of the pellet population. Next, the macroscopic results obtained in the shake flask measurement were assigned to an idealized, representative pellet. This pellet serves as a reference for analyzing the underlying mechanisms that control the production of labyrinthopeptin A1 and substrate consumption.

As part of the homogenization process, the entire cultivation medium is divided evenly according to the total number of the pellet $n_\textrm{p}$. It is assumed that each reference pellet interacts with the same surrounding medium. The equivalent volume

1 $$\begin{aligned} V_\textrm{mix,mean} = \dfrac{V_\textrm{mix,all}}{n_\textrm{p}} \end{aligned}$$

of the medium is defined based on the total volume $V_\textrm{mix,all}$ of entire culture medium in the shake flask. Following the approach introduced in Sect. “Cell counting”, the results of $n_\textrm{p}$ are summarized in Table 1.

**Table 1:** Estimated pellet population in 100 mL of medium on different cultivation days

*t* [h]	0	24	48	120	144
number of pellets [-]	289,791	4,738,125	5,706,140	6,867,207	7,329,000

Due to the rapid increase of the pellet population during the first 24 h of cultivation, the number of pellets was averaged after 24 h to estimate the pellet concentration at steady state. This resulted in a value of $n_\textrm{p} = 6.16 \times 10^{6}$ pellets per 100 mL culture and a averaged medium volume of $V_\textrm{mix,mean} = 1.62 \times 10^{7}$ *μ *m^3^ for each reference pellet.

The spatial mass transfer behavior between pellets and culture medium was described through the experimental determination of pellet size. The pellets were collected at the following stages of cultivation: 24, 72, 192, 216, 240 h. Analogous to the cell count method, the subsample of 2 mL diluted aliquot was analyzed optically using a digital microscope. Pellets with diameters smaller than 100 *μ *m or larger than 1000 *μ *m were excluded from the analysis, and the mean value $d_\textrm{p} = 186.37$ *μ *m was used as the constant radius of the reference pellet. The representative pellet volume is thus determined to be $V_\textrm{p} = 3.39 \times 10^{6}$ *μ *m^3^. This corresponds to a relative volume ratio of $\gamma _V = {V_\textrm{p}}/{V_\textrm{mix,mean}} = 20.88\%$. For a comprehensive overview of the modern methods for pellet detection, analysis, and counting, the reader is referred to Liu et al. [21].

### Kinetics model considering pellet growth, substrate consumption and product formation

The model approach proposed here is intended to numerically describe the consumption of oxygen (index: *\mathrm O*), glucose (index: *\mathrm S*), and glycerol (index: *\mathrm G*). The underlying mechanisms are represented by the following diffusion–reaction equations

2 $$\begin{aligned} \dfrac{\partial \boldsymbol{C} }{\partial t}= & \nabla \cdot (\boldsymbol{D} \nabla \boldsymbol{C} )-\boldsymbol{q} (\boldsymbol{C} , \alpha ), \end{aligned}$$

3 $$\begin{aligned} \dfrac{\partial C_\textrm{labA1}}{\partial t}= & f_\textrm{labA1}(\boldsymbol{q} , \alpha ), \end{aligned}$$

4 $$\begin{aligned} \dfrac{\partial \alpha }{\partial t}= & R_{\alpha }(\boldsymbol{C} , \alpha ). \end{aligned}$$

Here, the concentration of the various substrates, denoted by $\boldsymbol{C} = [C_\textrm{O}, C_\textrm{S}, C_\textrm{G}]^T$ is governed by diffusion term $\nabla \cdot (\boldsymbol{D} \nabla \boldsymbol{C} )$ and source term $\boldsymbol{q} =[q_\textrm{O}, q_\textrm{S}, q_\textrm{G}]^T$, as shown in Eq. (2). The production of labyrinthopeptin A1, expressed by its concentration $C_\textrm{labA1}$, is described by the evolution function $f_\textrm{labA1}$ in Eq. (3), which in turn depends on the pellet activity *α *, cf. Eq. (4).

With regard to the diffusion term $\boldsymbol{D}$, the heterogeneous hyphal structure of pellets can be characterized by experimental analyses using *μ *-CT [31, 39, 41–43], and the resulting inhomogeneous diffusivity has been demonstrated in the recent studies using oxygen profiling techniques [20, 31, 42]. These results of the morphology and diffusion analyses show a close relationship between structural porosity and diffusivity, with diffusivity decreasing toward the core of the pellet. As stated in the studies by Schmideder et al. [39] and Schmideder et al. [41], the effective diffusivity reads

5 $$\begin{aligned} D_{i,\textrm{eff}} =D_{i,\textrm{bulk}}\cdot k_\textrm{eff} {\;\;\textrm{with}}\;\;k_\textrm{eff}=(1-c_\textrm{h})^\beta {\;\;\textrm{for}}\;\;i=\textrm{O}, \textrm{S}, \textrm{G}. \end{aligned}$$

**Fig. 2:**
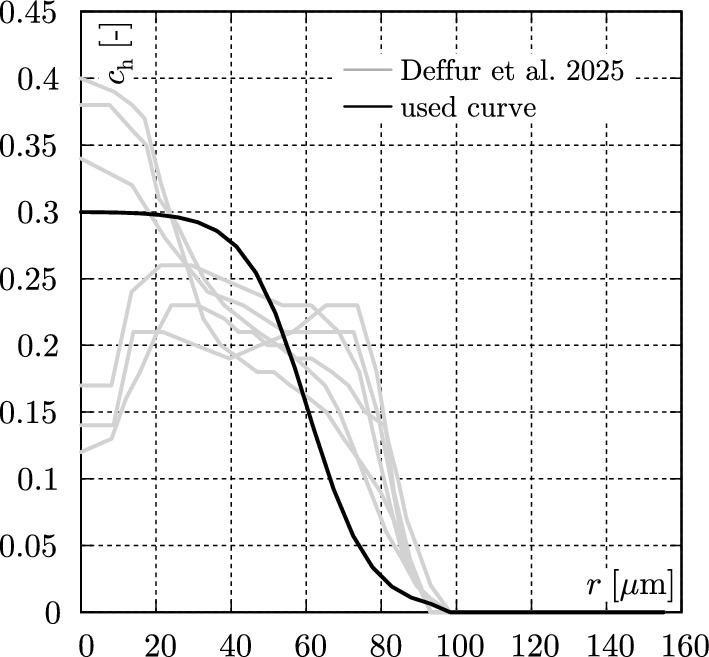
Radial hyphal distribution across the pellet and culture medium

Herein, $D_{i,\textrm{eff}}$ is the bulk diffusion coefficient and $k_\textrm{eff}$ denotes the correction function, which depends on the hphal fraction $c_\textrm{h}$ and the structural parameter *β *, describing nonlinear influences. According to the results of *μ *-CT analyses [31], the hyphal fraction

6 $$\begin{aligned} c_\textrm{h}(r_\textrm{p}) = c_\textrm{h,max} - \dfrac{(c_\textrm{h,max} - c_\textrm{h,min})}{1 + e^{-\eta (r_\textrm{p} - r_\textrm{p, mid})}} \end{aligned}$$

is described as an empirical function of the radial coordinated $r_\textrm{p}$ within a pellet, where the range of hyphal fraction is defined by $c_\textrm{h,min}$ and $c_\textrm{h,max}$ and its nonlinear profile is governed by the midpoint $r_\textrm{p, mid}$ and the steepness parameter *η *. The aim of this study is to provide a comprehensive description of the continuous radial distribution of the hyphal fraction within a pellet. Therefore, based on existing studies with *Aspergillus niger* strains SKAn1015 (regular branching), and MF22.4 (hyperbranching) [31, 42, 43], the highest hyphal fraction at the center $c_\textrm{h, max}$ is between 0.1 and 0.4, while the hyphal fraction at the periphery decreases to zero. In this study, a minimum value of $c_\textrm{h,min} = 0.001$ is assumed, taking into account the AESD. Regarding the nonlinear profile, an inflection point of $r_\textrm{p, mid} = 1/2\cdot 0.3\cdot d_\textrm{p}$ was applied, based on the measured hyphal fractions [31]. The corresponding hyphal distribution and the fitting results are shown in Fig. 2.

Here, the gray curves are taken from the six exemplarily selected pellets in Deffur et al. [31], and the hyphal distributions were rescaled to 93.18 *μ *m to match the pellet radius in this study. The assumed curve is shown in black and lies within the target range of the curves taken from the literature. Due to a lack of experimental results and for the sake of simplicity, this study assumes linear diffusion behavior, so that the effective diffusion coefficient remains constant for the given hyphal fraction and is not influenced by local substrate concentrations. Furthermore, it is hypothesized that the inhomogeneous hyphal structure has analogous effects on the effective diffusivity, a phenomenon that is addressed by the same correction coefficient $k_\textrm{eff}$. It can therefore be concluded that the diffusion tensors $\boldsymbol{D} = [D_\textrm{O, eff}, D_\textrm{S, eff}, D_\textrm{G, eff}]^T$ in Eq. (2) are reduced to scalar effective diffusion coefficients $D_{i, \textrm{eff}}$ for the substrate $i=\textrm{O}, \textrm{S}, \textrm{G}$ under the assumption of isotropic diffusion. This reduction occurs when the model domain in Sect. “Design of the model domain” is simplified to one-dimensional polar coordinates.

Substrate consumption is modeled taking into account two primary mechanisms: Maintenance and growth. Recent numerical studies have demonstrated a direct relationship between maintenance consumption and biomass [28, 31, 37, 40]. However, the source term in Eq. (2) is expressed as a function of the equivalent pellet activity $(c_\textrm{h} \cdot \alpha )$. This formulation accounts for the varying substrate requirements of the pellets, which depend on their activity *α * in different physiological states during cultivation. Based on experimental investigations [20, 44], it is known that pellet size only grows until 72 h and then remains roughly constant before decreasing again towards the end of cultivation.

According to Rinas et al. [29], the respiratory maintenance consumption rate of glucose, glycerol, and oxygen can be expressed as

7 $$\begin{aligned} q_\textrm{mS}= & k_\textrm{mS} \cdot (c_\textrm{h} \cdot \alpha ) \cdot M_\textrm{O} \cdot M_\textrm{S} \end{aligned}$$

8 $$\begin{aligned} q_\textrm{mG}= & k_\textrm{mG}\cdot (c_\textrm{h} \cdot \alpha ) \cdot M_\textrm{O} \cdot M_\textrm{G} \cdot f_\textrm{sw}^\textrm{SG} \end{aligned}$$

9 $$\begin{aligned} q_\textrm{mO}= & (1.07~q_\textrm{mS}+1.22~q_\textrm{mG} )\nonumber \\ & \quad \cdot f_\textrm{sw}^{\alpha } + k_\textrm{others} \cdot \left( c_\textrm{h} \cdot \alpha \right) \left( 1-M_\textrm{G}\right) \end{aligned}$$

to account for the preferred uptake of the pellet. Herein, the maximum specific consumption rates of glucose and glycerol are denoted by $k_\textrm{mS}$ and $k_\textrm{mG}$, respectively. The condition-dependence of the substrate uptake, is described by the Monod term

10 $$\begin{aligned} M_i = \dfrac{C_i}{C_i + C_{i,\textrm{crit}}} {\;\;\textrm{for}}\;\;i=\textrm{O}, \textrm{S}, \textrm{G}, \end{aligned}$$

where $C_i$ and $C_{i,\textrm{crit}}$ denote the present concentration and the critical concentration of each substrate, respectively. This term captures the dependence of uptake on substrate concentration in a saturation-type manner. Furthermore, glucose is preferred when it is abundant, while glycerol uptake dominates when the Monod term $M_\textrm{S}$ decreases as soon as the glucose supply is insufficient and the Monod-type term is close to zero. The transition between glucose to glycerol uptake (SG), which represents the diauxic effect, is determined by the function

11 $$\begin{aligned} f_\textrm{sw}^\textrm{SG} = e^{-{C_\textrm{S}}/{C_\mathrm{S_0}}}, \end{aligned}$$

which depends on the switching parameter $C_\mathrm{S_0}$ and the current glucose concentration $C_\textrm{S}$. This function reflects the diauxic behavior of microorganisms, in which the utilization of one carbon source takes precedence over the subsequent utilization of another. The exponential form in Eq. (11) indicates a smooth transition between glucose and glycerol when the glucose concentration $C_\textrm{S}$ approaches zero, thus avoiding sudden changes. If higher glucose concentrations are observed in the first three days, the switching function approaches zero and glucose becomes the predominant consumption resource. After glucose depletion in the following days, the switching function approaches one, shifting metabolic dominance toward glycerol. Accordingly, this formulation describes the transition from the glucose-driven metabolism at the start of cultivation to glycerol-supported metabolism at steady-state under glucose-limited conditions. The impact of oxygen limitation on the uptake rates of glucose and glycerol is expressed by a Monod-type term $M_\textrm{O}$. The total oxygen maintenance rate $q_\textrm{mO}$ is determined by the combined oxidation demand of glucose, glycerol, and other oxygen-dependent metabolic processes. The associated oxidation processes are given by

12 $$\begin{aligned} \textrm{C}_6\textrm{H}_{12}\textrm{O}_6 + 6\textrm{O}_2 \;\rightarrow \; 6\textrm{CO}_2 + 6\textrm{H}_2\textrm{O}&\;\;\;\;\;\;\;\;\; \text {for glucose}, \end{aligned}$$

13 $$\begin{aligned} \textrm{C}_3\textrm{H}_8\textrm{O}_3 + 3.5\textrm{O}_2 \;\rightarrow \; 3\textrm{CO}_2 + 4\textrm{H}_2\textrm{O}&\;\;\;\;\;\;\;\;\; \text {for glycerol}. \end{aligned}$$

These reactions show that 6 and 3.5 mol of $\textrm{O}_2$ are required per mole of glucose and glycerol, respectively, thereby establishing the foundation of the coefficients 1.07 and 1.22 in Eq. (9). An additional switching function

14 $$\begin{aligned} f_\textrm{sw}^{\alpha }=0.5(1+\tanh (9.8-10\alpha )) \end{aligned}$$

is introduced into Eq. (9) to regulate the onset of labyrinthopeptin A1 biosynthesis. This formulation reflects that part of the available carbon sources is diverted to the production of secondary metabolites once the pellet has reached its mature state. The coefficient $k_\textrm{others}$ in Eq. (9) takes into account oxygen consumption under carbon starvation conditions, as some experimental studies have shown that oxygen uptake still occurs even when carbon sources are limited [20, 45, 46].

To account for the growth kinetics, following associated specific growth rates we introduce

15 $$\begin{aligned} q_\textrm{gS}= & k_\textrm{gS}\cdot (c_\textrm{h} \cdot \alpha ) \cdot M_\textrm{O} \cdot M_\textrm{gS} \cdot f_\textrm{lim} \end{aligned}$$

16 $$\begin{aligned} q_\textrm{gG}= & k_\textrm{gG} \cdot (c_\textrm{h} \cdot \alpha ) \cdot M_\textrm{O}\cdot M_\textrm{gG}\cdot f_\textrm{lim}\cdot f_\textrm{sw}^{\alpha } \end{aligned}$$

17 $$\begin{aligned} q_\textrm{gO}= & 1.07~q_\textrm{gS}+1.22~q_\textrm{gG}, \end{aligned}$$

where the maximum growth-induced consumption values of glucose and glycerol for a given equivalent pellet activity are denoted by $k_\textrm{gS}$ and $k_\textrm{gG}$, respectively. Accordingly, the oxygen consumption associated with growth is defined in Eq. (17) analogous to the formulation of the oxygen maintenance rate using the identical coefficients 1.07 and 1.22 in Eq. (9). In context with the mechanism of substrate-limited growth mechanism, the modified Monod functions

18 $$\begin{aligned} M_{\textrm{g}i} = \dfrac{C_i - C_{i,\textrm{crit}}}{K_i + C_{i}} {\;\;\textrm{for}}\;\;i= \textrm{S}, \textrm{G} \end{aligned}$$

are integrated into Eqs. (15) and (16) by introducing the Monod constants $K_i$, which represents the substrate affinity in the growth kinetics, and the critical concentration $C_{i,\textrm{crit}}$. This ensured that the escalation in metabolic activity is stopped when the substrate supply is no longer sufficient to maintain anabolic processes. These modified Monod functions have been shown to establish a clear physiological priority: The maintenance process, which is considered the dominant metabolic activity, remains active even when substrate supply falls below the critical threshold $C_{i,\textrm{crit}}$, while the growth mechanisms are selectively deactivated [47–49].

In addition, the growth mechanism is restricted by activity-dependent limitations

19 $$\begin{aligned} f_\textrm{lim} = \alpha (1-\alpha ), \end{aligned}$$

which curtails the growth rate when the local activity *α * is inadequate or excessive. In contrast to the modified Monod functions, which relies on critical concentration conditions, this formulation describes the trade-off between growth and maintenance from a mechanistic perspective, taking into account metabolic load, especially for the hyphal network, where activity is elevated. It is therefore hypothesized that the hyphae may experience rapid local substrate depletion or an accumulation of inhibitory by-products. These factors could potentially lead to negative feedback on the growth mechanism.

Further, the same function $f_\textrm{sw}^\textrm{SG}$ as in Eq. (8) is integrated into Eq. (16) to regulate the selective consumption of glucose and glycerol.

Additionally, a source term is included for maintenance and growth kinetics to account for oxygen supply under shaking conditions. This approach differs from the recent studies [24, 28, 40, 42] that applied a Dirichlet boundary condition. This condition is also not applicable here, as it assumes invalidly a fixed concentration at the boundary of each domain for shake flask cultures. Moreover, the gas-liquid interface and mixing patterns vary dynamically with orbital motion, resulting in time-dependent oxygen transfer that cannot be captured by a Robin boundary condition either. As shown by Kuschel and Takors [50], there is a close relationship between flow rate and gas volume, confirming the inhomogeneous distribution of dissolved oxygen. Thus, a source term formulation

20 $$\begin{aligned} q_\textrm{f, BC} = k_{L}a (C^{\infty } - \bar{C}_\textrm{O}) \end{aligned}$$

was applied to the fluid domain, where the supply rate in the fluid domain $q_\textrm{f, BC}$, also termed as volumetric oxygen transfer rate, depends on the volumetric mass transfer coefficient $k_{L}a$, the saturated dissolved oxygen concentration in the fluid $C^{\infty }$, and the averaged concentration in the fluid domain $\bar{C}_\textrm{O}$. Within the homogenized framework, this formulation considers both the $k_{L}a$ defined on a lab scale and the periodic motion of the fluid elements in the shake flask. According to the results of recent experimental studies, the $k_{L}a$ values obtained under conditions of a working volume of 100 mL in 500 mL shake flasks operated on an orbital shaker with a shaking amplitude of 50 mm and a shaking frequency of 180 rpm are approximately *36.5~± ~5* h^-1^. This is representative value of the conditions in a laboratory-scale bioreactor.

In accordance with the previously formulated mechanism of maintenance and growth, the temporal evolution of the substrates

21 $$\begin{aligned} q_i= & q_{\textrm{m}i} + q_{\textrm{g}i}, {\;\;\textrm{for}}\;\;i = \textrm{S}, \textrm{G} \end{aligned}$$

22 $$\begin{aligned} q_\textrm{O}= & q_\textrm{m O} + q_\textrm{gO} - q_\textrm{f, BC} \end{aligned}$$

integrates the contributions of both the maintenance and growth mechanisms and the oxygen supply.

As a function of the consumption of different substrates, the temporal evolution of pellet

23 $$\begin{aligned} R_\alpha = \dfrac{k_{\alpha }}{c_\textrm{h}}(0.14q_\textrm{gS} + 0.16q_\textrm{gG}) \end{aligned}$$

in Eq. 4 is expressed as a linear combination of the two substrate-specific consumption rates. This formulation assumes that the maintenance mechanism makes a negligible contribution to the increase in activity. In this modeling approach, the coefficients 0.14 and 0.16 represent the stoichiometric contribution of glucose and glycerol, respectively, to the supported growth. These values are derived by converting the molar Adenosine triphosphate (ATP) yield from complete substrate oxidation to a per-gram basis using the corresponding molar masses. In *Escherichia coli* in particular, the complete oxidation of 1 mol of glucose theoretically yields a maximum of 26 mol of ATP, while 1 mol of glycerol yields up to 15 mol of ATP [51]. The effective growth rate is scaled by a kinetic parameter $k_{\alpha }$ and additionally normalized by the local hyphal fraction $c_\textrm{h}$. This formulation suggests that, with identical substrate utilization, hyphal networks with higher $c_\textrm{h}$ exhibit a comparatively lower increase in activity, while those with lower $c_\textrm{h}$ can show a more pronounced increase in pellet activity.

After determining the substrate consumption rates, the formulation for the production of labyrinthopeptin A1 is modeled as a process dependent on the rate of carbon source consumption

24 $$\begin{aligned} f_\textrm{labA1} = \eta _\textrm{labA1}(0.75~q_\textrm{mS} + 0.73~q_\mathrm{m{G}})(1-f_\textrm{sw}^{\alpha }). \end{aligned}$$

This indicates that the production rate is proportional to both glucose and glycerol uptake rates and to the metabolic maturation of the pellet activity. Since labyrinthopeptin A1 consists predominantly of hydrophobic amino acids [52], the carbon skeletons of these residues originate from the main carbon sources provided in the culture medium, in this study are glucose and glycerol. The coefficients 0.75 and 0.73 are derived from stoichiometric conversion factors that relate glucose and glycerol utilization to the theoretical carbon yield for the biosynthesis of labyrinthopeptin A1. As reported by Meindl et al. [53], labyrinthopeptin A1 was identified by mass spectrometric analysis with the molecular formula C_92_H_119_N_23_O_25_S_4_. In Eq. (24), $\eta _\textrm{labA1}$ denotes the production conversion efficiency, which reflects that the biosynthesis of secondary metabolites proceeds with a characteristic yield and that labyrinthopeptin A1 is not the only product formed [52]. Since growth arrest is an important signal for triggering secondary metabolism, an additional term is introduced that depends on pellet activity. Here, the production term is activated by a switching function $f_\textrm{sw}^{\alpha }$ when *α * approaches maturity. This threshold value suggests the assumption that labyrinthopeptin A1 is produced under conditions of high activity. Following these events, the formation of labyrinthopeptin A1 is physiologically limited to hyphal networks with sufficient activity, thereby preventing premature or non-physical production under conditions of low metabolism. In summary, the model is formulated in terms of substrate consumption rates and takes into account both growth-related consumption and maintenance metabolism. In addition, it captures the dynamics of pellet growth and product formation.

### Selection of diffusivity parameters

Several studies have hypothesized that essential nutrients, such as dissolved oxygen and glucose, are transported exclusively by molecular diffusion [25, 28, 29, 31, 35, 36, 38, 40, 54]. It is assumed that the effective diffusion coefficient $D_\textrm{eff}$ within the pellet depends on the pellet’s void fraction. A detailed description and overview of the effective diffusion coefficient can be found in Schmideder et al. [39], which takes into account the molecular diffusion coefficient, tortuosity, and porosity. Several studies in the existing literature have found a simple linear relationship between $D_\textrm{eff}$ and $D_\textrm{bulk}$, $D_\textrm{eff} =D_\textrm{bulk} (1-c_\textrm{h})$ [25, 28, 29, 35, 40], where $D_\textrm{bulk}$ denotes the maximal molecular diffusion coefficient. However, one study has proposed an alternative hypothesis [24]. This hypothesis states that, under stirred conditions, $D_\textrm{eff}$ can reach 5 to 10 times the diffusion coefficient of a liquid $D_\textrm{bulk}$. This phenomenon occurs due to turbulence, which is characterized by fluctuations in local volume density. These fluctuations occur once the local volume density reaches a critical value [24]. Additionally, several studies have included an exponent *β * to account for the complex pore geometries characteristic of pellets [31, 39, 41]. Empirical findings have shown that this exponent’s value generally tends towards 2 [31, 39, 41]. Recorded volume diffusion coefficients for oxygen range from $D_\textrm{O,bulk}=6.08 \times 10^{-6}$ to $10.44 \times 10^{-6}$ m^2^ h^-1^ in the temperature range of 20 to $40^{\circ }$C [24, 25, 28, 29, 35]. Earlier studies found the diffusion coefficients for glucose and glycerol to be in the range of $8.2 \times 10^{-7}$ to $3.24 \times 10^{-6}$ m^2^ h^-1^ [29, 36, 54] and for $D_\textrm{G, bulk}$ between $7.3 \times 10^{-7}$ and $3.5 \times 10^{-6}$ m^2^ h^-1^ [55]. In the present study, the diffusion coefficients for each substrate were selected within these reported ranges, as shown in Table 2.

### Model implementation and numerical conditions

#### Numerical implementation of different cases

The reaction-diffusion equations for substrate consumption and labyrinthopeptin A1 production were solved numerically. As described above, the spatial domain was uniformly discretized with an element size of 5 *μ *m. The problem was then converted into a system of coupled ordinary differential equations that track the temporal evolution of substrate, pellet activities, and product concentration at each spatial node. Time integration was performed using the ordinary differential equation solver ODE15s (Matlab^®^ R2024a, The MathWorks, Inc.), which is suitable for stiff systems arising from nonlinear reaction kinetics and diffusion. A time step of $1\times 10^{-4}$ h was used throughout the simulated cultivation period. Assuming axisymmetric behavior, the model was reduced to a one-dimensional diffusion–reaction problem with five field variables in radial coordinates. The one-dimensional domain was segmented into two distinct regions representing the hyphal pellet and the surrounding fluid medium.

In order to calibrate the model with the experimental results, two main scales are considered in this study: (i) Cultivation in the shake flask and (ii) oxygen profiling in the diffusion setup, combined with different pellet conditions, see Fig. 1. The pellets taken for oxygen profiling exhibited different states on different days. In addition to these changing states, it is essential to consider further states shifting impacted by the preparation time $\Delta t_\textrm{prep}$ and the duration of the experiment. In addition, a range of domain sizes was used, taking into account the oxygen supply conditions.

In the shake flask simulations, a pellet-fluid length ratio of $\gamma _L = 59.34$% was adopted, which corresponds to the estimated volume ratio $\gamma _V$ introduced in Sect. “Kinetic modeling of filamentous pellets”. This results in 19 and 12 evenly distributed spatial grid nodes for the hyphal and fluid domains, respectively. In this case, all substrates except labyrinthopeptin A1 were evaluated exclusively within the fluid phase, and their spatial average was used for comparison with the experimentally monitored concentrations, see $\bar{C}_i$ in Fig. 1. For labyrinthopeptin A1, the total concentration in both the pellet and fluid domains was considered. Regarding the boundary conditions for glucose and glycerol, Neumann boundary conditions ($\partial C_i / \partial r = 0$) were imposed at the outer domain boundary to reflect the absence of external flux of carbon substrates from the surrounding fluid or gas phase occurs during the process.

With regard to oxygen profiling, the measurement starts at a distance of approximately 200 *μ *m from the surface of the pellet, featuring an empirically determined average radius of 150 *μ *m. Consequently, a length ratio of $\gamma _L = 75$% is adopted to match the fluid domain present in the experimental configuration of oxygen profiling. Due to the difference in pellet size between shake flask and oxygen profiling simulations, the number of grid nodes remains constant while the element size is modified to 8.33 *μ *m. This spatial scaling accounts for the difference in pellet size between the two scales. Additionally, different boundary and initial conditions were specified for the cultivation and oxygen profiling simulations. Considering the diluted medium, the glucose and glycerol concentrations were set to 20% of their initial values. Based on the cultivation simulations, the simulated internal states at certain time points (48 and 168 h) served as the initial conditions at $t_0$ for the oxygen profiling simulations, and the concentration profiles within the pellet were retained through the aforementioned spatial mapping, see also Fig. 1. Additionally, a preliminary simulation was performed for $\Delta t_\textrm{prep}=10$ min in combination with the surrounding fluid domain, depending on the predefined boundary conditions (50% and 100% saturation levels), considering the handling time before recording the concentrations. The resulting field variables across the entire domain from the simulation with a waiting/preparation time of $\Delta t_\textrm{prep}=10$ min were applied as the initial conditions for the subsequent oxygen profiling simulations. With respect to the boundary conditions, Dirichlet boundary conditions were applied to glucose and glycerol to ensure a continuous oxygen supply through the Stokes flow. Regarding the oxygen supply, a Neumann boundary condition was implemented that considers the boundary layer at the pellet surface and the associated limited oxygen supply in the radial direction.

#### Parameter identification

The model was calibrated using experimental data obtained from the offline analyses described in Sect. “Cultivation and offline analysis” and the oxygen profiling experiments expounded in Sect. “Oxygen microprofiling measurements”. The model parameters were identified as part of an inverse analysis by minimizing the error between experimentally measured data and numerically simulated results. The offline measurements provided macroscopic information in the form of time series, which were then compared with spatially averaged or integrated quantities derived from the cultivation simulation. In contrast, the oxygen profiling experiments provide spatiotemporal data at the microscopic level, which are directly compared with the corresponding field variables obtained from the simulations with different boundary and initial conditions. In summary, the parameter identification was performed by simultaneously optimizing the agreement between simulations and experiments across two scales: (i) The first part of the study at lab scale, see Fig. 1a, deals with the averaged time-dependent substrate consumption and the production of labyrinthopeptin A1 during cultivation in the shake flask. (ii) The second part at pellet scale, see Fig. 1b, consists of four oxygen profiling experiments performed under higher and lower oxygen concentrations on pellets collected after 48 h and 168 h of cultivation, respectively. These two scales result in a total of 5 distinct scenarios.

It is important to note that *α * is an internal model variable that cannot be directly quantified experimentally. Therefore, the parameters that control the activity development in Eq. (23) were identified indirectly by their coupling with four other experimentally observable quantities.

Furthermore, when comparing the result with oxygen profiling data, the influence of the oxygen sensor manipulation must be considered. Due to the stepwise measuring method described in Sect. “Oxygen microprofiling measurements”, the measured oxygen concentrations represent a temporally evolving field rather than an instantaneous state. To remedy this issue, the simulated oxygen profiles were synchronized with the experimental measurement protocol over time. The movement of the sensor tip was synchronized with the start of the profiling experiment, denoted as $t_1$ after the pre-handling for $\Delta t_\textrm{prep}$, and for each specified sampling time $t_i$, the sensor position $r_i$ was estimated by applying a predefined penetration velocity of *v = 0.667* *μ *m s^-1^. The corresponding oxygen concentration was estimated by interpolation between the simulated values of the two nearest spatial nodes of the hyphal domain. This procedure ensured a consistent and temporally accurate comparison between experimental measurements and simulation results.

**Table 2:** Model parameters

Parameter	Unit	Value	Source
*Morphological parameters*			
$d_\textrm{p}$	*μ *m	186.37	Measured
$r_\textrm{p}$	*μ *m	98.18	Measured
$c_\textrm{h, max}$	-	0.3	[31]
$c_\textrm{h, min}$	-	0.001	[31]
$\gamma _V$	%	20.88	Measured
$\gamma _L$	%	59.34	Measured
*η *	m^-1^	91429	Measured
*Initial cultivation conditions*			
$\alpha _0$	-	0.1	Chosen
$C_{\textrm{O}_0}$	g/L	7.6*× *10^-3^	Measured
$C_{\textrm{S}_0}$	g/L	8.69	Measured
$C_{\textrm{G}_0}$	g/L	10.22	Measured
$k_La$	h^-1^	40	Optimized
*Kinetic parameters*			
$D_\textrm{O,bulk}$	m^2^ h^-1^	8.7*× *10^-6^	[29, 31]
$D_\textrm{S,bulk}$	m^2^ h^-1^	2.2*× *10^-6^	[24, 29]
$D_\textrm{G,bulk}$	m^2^ h^-1^	3.5*× *10^-6^	[55]
*β *	-	1.76	[31, 41]
$k_\textrm{mS}$	g/(Lh)	3.5	Optimized
$k_\textrm{mG}$	g/(Lh)	5	Optimized
$k_\textrm{other}$	g/(Lh)	1	Optimized
$k_\textrm{gS}$	g/(Lh)	300	Optimized
$k_\textrm{gG}$	g/(Lh)	80	Optimized
$K_\textrm{S}$	g/(Lh)	1	Chosen
$K_\textrm{G}$	g/(Lh)	1	Chosen
$C_\textrm{S, crit}$	g/(Lh)	0.01	[24]
$C_\textrm{G, crit}$	g/(Lh)	0.01	Chosen
$C_\textrm{O, crit}$	g/(Lh)	1.9*× *10^-3^	Chosen
$k_{\alpha }$	g/(Lh)	1.5*× *10^-2^	Optimized
*α *	-	[0 1]	Chosen
$\eta _\textrm{labA1}$	%	3.75	Optimized

The consumption rates of glucose, glycerol, and oxygen were calibrated using the four oxygen profiling experiments. In these experiments, substrate consumption was negligible, as a continuous nutrition supply of 20% kept external concentrations at a constant level. The metabolite activity of the pellets exhibited fluctuations between 48 h and 168 h. After 48 h of cultivation, the pellets demonstrated a significant increase in their growth rate. During this phase, the oxygen consumption rate was primarily due to metabolic processes related to growth. After 168 h of cultivation, the pellets had reached a mature state, and oxygen uptake was primarily driven by maintenance metabolism. The optimized parameter values were then implemented in the first scenario of the shake flask cultivation, allowing a direct comparison between simulated concentration profiles and experimentally measured concentrations. Adjustments were then made to refine the parameters and identify a set of parameters that could accurately describe all five scenarios mentioned above. A summary of the parameter values required for the calculation is shown in Table 2.

### Case studies: factors influencing cultivation performance

#### Influence of $k_La$ on cultivation process

As oxygen limitation can lead to growth stagnation and product inhibition [29], the volumetric oxygen transfer coefficient $k_La$ plays a pivotal role in pellet cultivation. In the context of pellet cultivation in bioreactors, it is known that $k_La$ is influenced by hydrodynamic factors, including stirring and aeration rate, fluid viscosity, and the geometric configuration of the shake flask [56]. In the present study, the cultivation process was carried out at a constant shaking frequency of 180 rpm. According to the simulation results reported in Kardooni et al. [56], the value of $k_La$ increases remarkably with increasing shaking frequency. Therefore, this case study systematically varies $k_La$ to investigate how changes in shaking frequency affect oxygen transfer. Subsequently, the impact of these changes on pellet development and cultivation efficiency is examined. This methodological approach enables a comprehensive understanding of the complex relationship between physical transport phenomena and biological responses. The experimentally determined $k_La$ of *36.5~± ~5* h^-1^ defines the parameter as a series of discrete values: 25, 35, and 45 h^-1^. The set of parameters considered represents a realistic range of oxygen transfer conditions typically encountered in shake flasks.

#### Impact of volume partition on the cultivation process

In this study, the relative volume ratio, which represents the ratio of pellet volume to available nutrient supply volume, is $\gamma _V=20.88$%. A higher value of the ratio indicates an increase in the nutrient volume relative to the pellet, which improves the effective substrate supply. For the reference case, $\gamma _V$ is determined based on the experimentally measured average pellet diameter, assuming that the working volume is evenly distributed across each pellet and that deviations in pellet size are negligible.

**Fig. 3:**
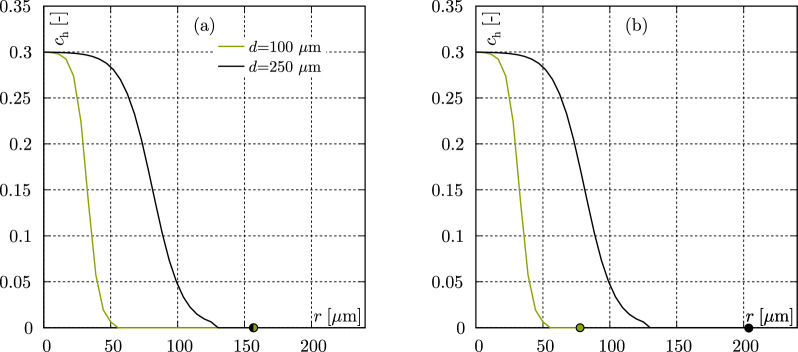
Radial hyphal distributions across pellets with various diameters (*d=100/250* *μ *m) and the surrounding culture medium: **a** Medium volumes of $\gamma _v=79.59$% (*d=100* *μ *m) and $\gamma _v=31.83$% (*d=250* *μ *m) and **b** $\gamma _v=22.76$% for both diameters

In the first case series of this section, the fluid domain allocated to each pellet remains the same as in the reference case. Two additional pellet diameters (100 *μ *m and 250 *μ *m) are considered to investigate the effect of pellet size on growth kinetics and productivity. Next, relative volume ratios of 79.59% and 31.83% are applied, and the corresponding radial hyphal distributions across the pellet and surrounding culture medium are shown in Fig. 3a.

The second case study assumed the same total amount of pellets and medium volume, but accounts for the pellet size distribution throughout the cultivation system by applying the probability density function [56]

25 $$\begin{aligned} p(d_\textrm{p})=\dfrac{\alpha _2}{\alpha _1}\left( d_\textrm{p}-50\right) ^{\alpha _2-1}\exp \left( -\dfrac{(d_\textrm{p}-50)^{\alpha _2}}{\alpha _1}\right) , \end{aligned}$$

using a working volume of 100 mL. However, the probability density function obtained on different cultivation days has different parameter values. In line with the above considerations, the present study uses the mean values of $\alpha _1$ and $\alpha _2$ with respective values of 56 and 0.89, resulting in a relative volume ratio to $\gamma _V=22.76$%. As in the first case study, pellets with diameters of 100 and 250 *μ *m are examined, and the radial hyphal distributions corresponding to the pellet and the surrounding culture medium are shown in Fig. 3b.

### Extended model considering additional substrates

The proposed model can be extended to understand different cultivation protocols. Based on the experimental study by Kozanecka et al. [20], the model was applied to cultivate the filamentous bacterial strain *A. namibiensis* (DSM 6313) to quantitatively analyze the difference between unsupplemented (control culture) and salt-supplemented cultures. However, the oxygen profiling results of Kozanecka et al. [20] are not considered due to the absence of the paired oxygen profiling data and the corresponding morphological information required in Sects. “Cultivation and offline analysis” and “Oxygen microprofiling measurements”, as well as the lack of the necessary domain design. First, the model is adjusted to fit the temporal profiles of substrates and labyrinthopeptin A1 based on the results of salt-supplemented cultures. Next, further adjustments to the model parameters are applied to fit the results of the unsupplemented control culture. Comparing the obtained model parameters helps explain the underlying effects.

Considering the starch, which has a similar consumption rate to glucose and is therefore not shown, that was applied, the model is extended with a hydrolysis mechanism. Unlike the formulation in Eq. (2), starch concentration is modeled using a global approach that assumes starch is uniformly distributed throughout the entire culture volume due to macroscopic mixing. This approach also accelerates the simulation by solving relatively stiff differential equations, affected by the potentially very small diffusion coefficient of starch (index: *\mathrm st*). The hydrolysis rate

26 $$\begin{aligned} q_\textrm{st} = k_\textrm{hyd} \cdot (c_\textrm{h} \cdot \alpha ) \cdot f_\textrm{st} \end{aligned}$$

is assigned a hydrolysis coefficient $k_\textrm{hyd}$ and the kinetic function

27 $$\begin{aligned} f_\textrm{st} = \dfrac{C_\textrm{st}}{K_\textrm{st} + C_\textrm{st}} \cdot \dfrac{K_\textrm{I,st}}{K_\textrm{I,st} + C_\textrm{G}} \end{aligned}$$

considers saturation with respect to the global starch concentration $C_\textrm{st}$, which is controlled by the half-saturation constant $K_\textrm{st}$ and impacted by non-competitive inhibition. This inhibition is affected by the local glucose concentration $C_\textrm{G}$ and the inhibition constant $K_\textrm{I,st}$. The global rate is then averaged over all spatial points, modifying the consumption rate formulated in Eqs. (21, 22) as

28 $$\begin{aligned} q_\textrm{S} = q_\textrm{mS} + q_\textrm{gS} - 1.11q_\textrm{st}. \end{aligned}$$

This considers the ratio of the molecular weights of glucose (180.16 g mol^-1^) and soluble starch (162.14 g mol^-1^) as a monomeric unit. Among the parameters for hydrolysis kinetics, $K_\textrm{st}=0.4$ gL^-1^ was taken from the study by Heitmann et al. [57]. The other two strain-dependent parameters $k_\textrm{hyd}$ and $K_\textrm{I,st}$, as well as the other necessary parameters in the above model, will be adjusted to fit the experimental results. The parameters that changed in this application are summarized in Table 3.

**Table 3:** New parameters for model transfer on the salt-supplemented culture

Parameter	Unit	Value	Source
Initial cultivation conditions			
$C_{\textrm{st}_0}$	g/L	9.0	Measured
$k_La$	h^-1^	60	Optimized
Kinetic parameters			
$k_\textrm{mS}$	g/(Lh)	25	Optimized
$C_{\textrm{S}_0}$	g/L	3	Optimized
$k_\textrm{gS}$	g/(Lh)	650	Optimized
$k_\textrm{hyd}$	g/(Lh)	60	Optimized
$K_\textrm{st}$	g/L	0.4	[57]
$K_\textrm{I, st}$	g/L	1	Optimized
$\eta _\textrm{labA1}$	%	6.2	Optimized
$k_{\alpha }$	g/(Lh)	8.5*× *10^-3^	Optimized

Based on the model transfer and calibrated model parameters, the extended model was applied to the controlled culture. Due to significant differences observed in the glycerol profile and productivity after 72 h, only three parameters were modified, see Table 4.

**Table 4:** New parameters for model transfer on the unsupplemented control culture

Parameter	Unit	Value	Source
Kinetic parameters			
$k_\textrm{mG}$	g/(Lh)	1.2	Optimized
$k_\textrm{gG}$	g/(Lh)	10	Optimized
$\eta _\textrm{labA1}$	%	3	Optimized

## Results

### Shake flask cultivation of *Actinomadura namibiensis*

**Fig. 4:**
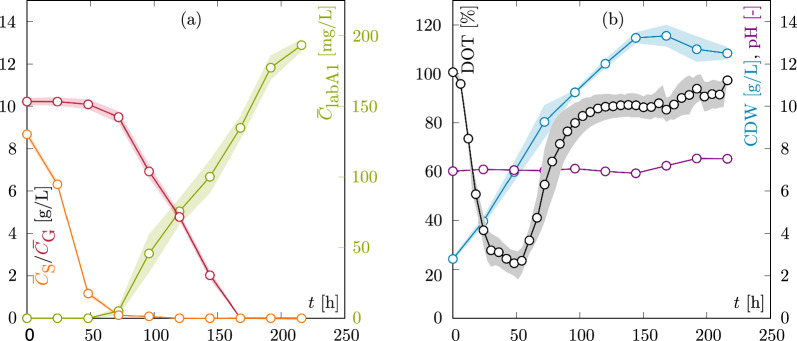
Experimental data from shake flask cultures of *A. namibiensis*, supplemented with 50 mM (NH_4_)_2_SO_4_ ($30^{\circ }$C, 180 rpm): **a** Concentrations of substrates and product and **b** cell dry weight, pH, and DOT. Shaded area represent the standard deviation and circles connected by lines indicate the mean values of the data. All data are means from four independent cultivations

A systematic approach was taken to assess the progress and stability of each shake flask culture. This involved measuring substrate, product and cell dry weight (CDW) concentrations, pH, and DOT. The resulting cultivation data are summarized in Fig. 4.

Previous studies have shown that glucose and starch are metabolized in almost identical ways [13, 20]. Therefore, only glucose was analyzed in the present study. Within the initial 72 h of cultivation, complete depletion occurred, after which glycerol metabolism continued until 168 h. Product synthesis began after 72 h, with cultures reaching a labyrinthopeptin A1 titer of *193.4~± ~4.2* mg/L on the final day of cultivation. CDW increased continuously until 144 h, when it reached a maximum of *13.3~± ~0.3* g/L. The pH remained stable at around 6.9 before rising to 7.5 in all four cultures after 144 h. DOT reached a low point of about 20% after 48 h and then rose to 80–90% after 96 h. This level remains constant between 129 and 168 h before increasing to 100% in the last three days of cultivation.

### Parameter estimation and simulation of cultivation system

**Fig. 5:**
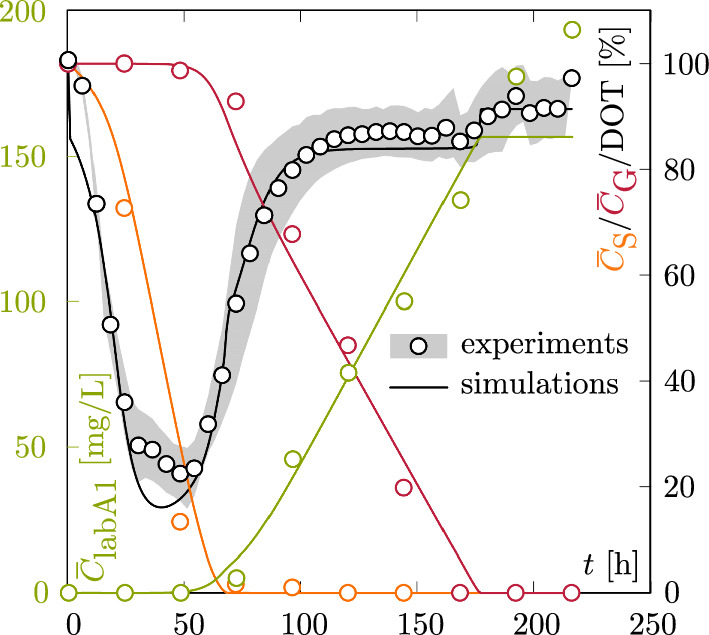
Comparison of simulation and experimental results of shake flask cultivation, with the simulation data plotted as lines and the mean experimental measurements shown as circles. The shaded area represented the standard deviation

The performance of the proposed model was evaluated by comparing the simulated profiles of glucose, glycerol, oxygen, and labyrinthopeptin A1 in the shake flask throughout the cultivation with the corresponding experimental measurements. The results of this comparison are shown in Fig. 5.

Overall, the model demonstrates a high degree of agreement with the observed data and effectively captures the prevailing trends in substrate consumption and product formation. The discrete markers with different shapes and colors represent the concentrations of glucose, glycerol, oxygen, and labyrinthopeptin A1, which were measured at 24 h intervals. The simulation results are plotted at 1 h intervals to allow for a smooth and continuous representation of the cultivation dynamics. During the first 48 h of cultivation, a slight deviation in the oxygen profile was observed. In the experiment, the minimum oxygen concentration was observed after 48 h of cultivation, whereas in the simulated profile, the minimum occurs between 24 and 48 h. In addition, a slight deviation in the labyrinthopeptin A1 profile was observed at the late phase of cultivation, accompanied by a shortage of the carbon source glycerol. The experimental data show that labyrinthopeptin A1 continues to accumulate even after glycerol has been fully consumed. However, according to the assumptions made in this study, no further production takes place in the simulation once the carbon source is exhausted.

**Fig. 6:**
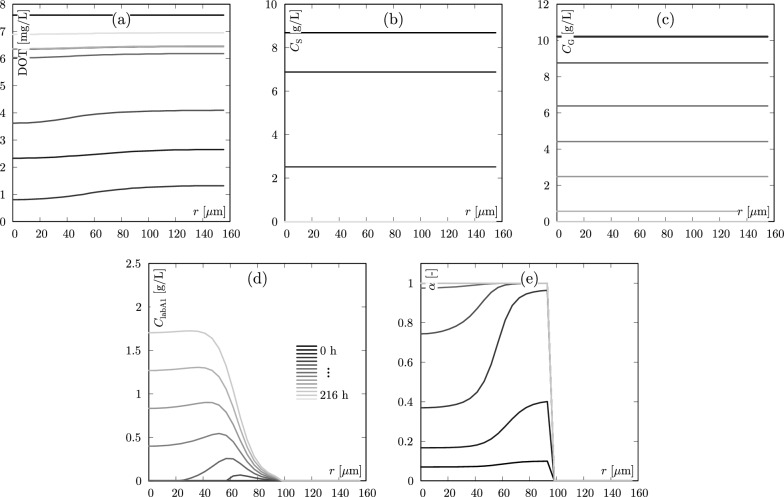
Simulation results of pellet and fluid configurations over the cultivation time: **a** DOT profiles, **b** glucose concentration profiles, **c** glycerol concentration profiles, **d** concentration distribution of labyrinthopeptin A1, and **e** pellet activity *α * profiles

Figure 6 shows the corresponding pellet and cultivation medium configurations, which provide detailed information about the spatial distribution of substrate nutrients, including oxygen, glucose, and glycerol, as well as the radial activity profile and product accumulation within the culture system over several days of cultivation.

It can be seen that the glucose and glycerol concentration profiles shift downward from their initial values to 0 over the course of cultivation. Conversely, the downward trend in the oxygen profile shows signs of recovery after 48 h of cultivation, as the oxygen supply exceeds the consumption rate of hyphae. Both the oxygen and activity profiles show their maximum values at the pellet boundary, while lower levels are observed inside the pellet. The activity profile increasingly shifts toward higher values and finally reaches a mature state after 96 h. Hyphae with lower volume fractions exhibit the fastest growth, and the peak value of *α * is consistently at the pellet boundary. The formation of labyrinthopeptin A1 is governed by *α * when $f_\textrm{sw}^{\alpha }$ has been activated. Accordingly, product accumulation begins at least 72 h after the start of cultivation.

The subsequent investigation of the oxygen profiles within the pellets, conducted on days 2 (48 h) and 7 (168 h), was performed in an oxygen profiling experiment under two different boundary conditions, to be 50% and 100% oxygen saturation in the liquid domain surrounding the pellet.

**Fig. 7:**
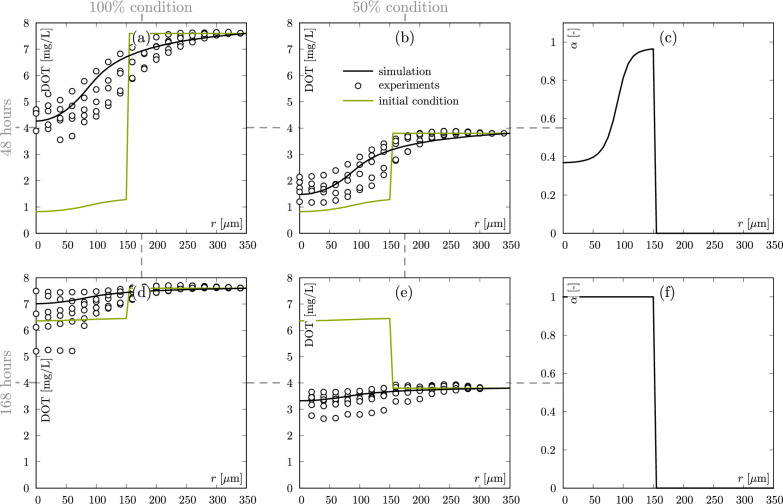
Oxygen profiling experiments and simulation results of pellet and fluid domains: Oxygen profiles of the pellet from 48 h of cultivation under **a** 100% and **b** 50% oxygen saturation. Pellet activity profiles from the **c** 48 h and **f** 168 h of cultivation. Oxygen profiles of the pellet on 168 h of cultivation under **d** 100% and **e** 50% oxygen saturation. The green lines show the oxygen profiles at $t_0$ before profiling. The black, bold curves show the simulated results, and the circles identify the experimental measurements used for comparison

The corresponding results are presented in Fig. 7. In general, the results show a good agreement between the simulated and experimentally measured oxygen concentrations.

After 48 h, the oxygen profile exhibits a lower concentration compared to 168 h, both before and after the oxygen profiling experiment under the 100% saturation. This indicated higher oxygen consumption and greater oxygen limitation in the early cultivation phase. This phenomenon is particularly evident in the center of the pellet, where the oxygen concentration increased from 0.82 to 4.26 mg/L after the profiling experiment after 48 h. After 168 h, a slight increase in oxygen concentration from 6.36 to 7.01 mg/L was observed. A similar trend can be observed under 50% saturation condition. The oxygen concentration in the center of the pellet increased from 0.82 to 1.47 mg/L after 48 h, whereas after 168 h, it decreased from 6.36 to 3.32 mg/L. This decrease is due to the lower external oxygen concentration, which drives oxygen diffusion out of the pellet.

### Substrate consumptions

**Fig. 8:**
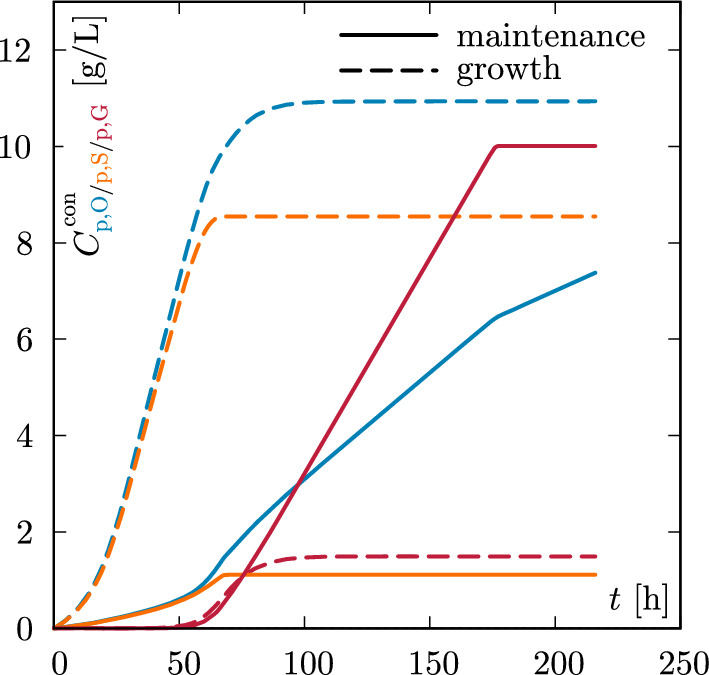
Simulation results of oxygen (blue), glucose (orange), and glycerol (red) consumption (index: con) $C^\textrm{con}_{\textrm{p},i}$, with $i=\mathrm{O/S/G}$ inside the pellet with respect to time

The cumulative substrate consumption of oxygen, glucose, and glycerol throughout the entire cultivation process is shown in Fig. 8. Consumptions attributed to pellet growth and pellet maintenance are shown with dashed and solid curves, respectively, while the different substrates are denoted with individual colors.

The model outputs reveal a clear hierarchy in carbon source utilization. Glucose primarily supports pellet growth, with total glucose consumption for growth exceeding that required for maintenance. Conversely, the glycerol uptake contributes less to pellet growth. Its uptake rate remains low as long as glucose is available. Glycerol consumption for both growth and maintenance begins when glucose is depleted, consistent with classical diauxic growth behavior. Microorganisms preferentially consume the carbon source that supports a higher growth rate. In the present study, the derived glucose uptake rate $k_\textrm{gS}$ is 3.75 times higher than the derived glycerol uptake rate $k_\textrm{gG}$. Following pellet maturation, the onset of labyrinthopeptin A1 production becomes evident. At this stage, the metabolic allocation of available carbon source shifts. The substrates are no longer directed only toward pellet evolution, but are instead partitioned between maintaining the mature pellet state and supporting secondary metabolite biosynthesis. Since the metabolism of *A. namibiensis* is predominantly based on oxidative reactions, oxygen acts as the essential terminal electron acceptor. As a result, oxygen consumption proceeds continuously throughout the entire cultivation period.

### Impacts of the volumetric oxygen transfer coefficient $k_La$ on cultivation process

**Fig. 9:**
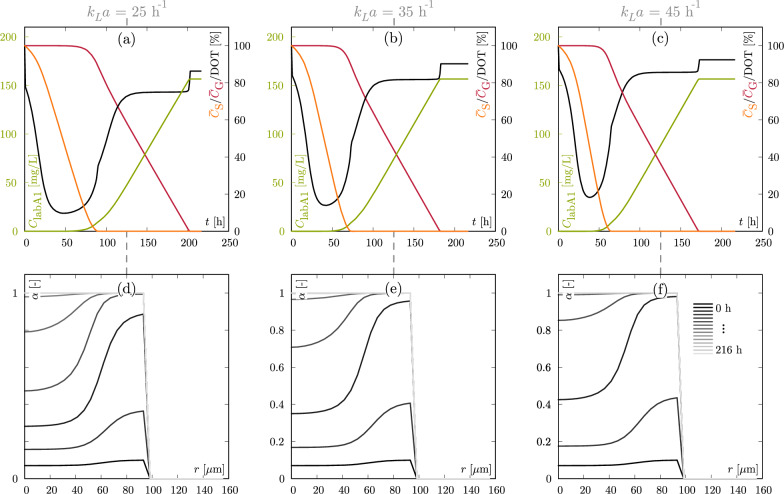
Simulation results of the pellet cultivation process under various hydrodynamic conditions with $k_La$ at: **a** and **d** 25 h^-1^, **b** and **e** 35 h^-1^, and **c** and** f** 45 h^-1^. Shown are the time-profiles of substrates and product, whereas the diagrams below illustrate the corresponding pellet states

Oxygen is the main limiting substrate in the cultivation process, and its insufficient availability can result to stagnation into pellet growth. To investigate this effect, the influence of external oxygen supply intensity, expressed as volumetric oxygen transfer coefficient $k_La$, on pellet development and productivity throughout cultivation is examined. The corresponding simulation results are shown in Fig. 9.

The top three graphs (a) to (c) illustrate the global concentrations of nutrients and product labyrinthopeptin A1 throughout the cultivation period, while the bottom three diagrams (d) to (f) show the corresponding pellet activity profiles at $k_La$ between 25 and 45 h^-1^. Although the final productivities of the three cases are comparable, clear differences in the temporal changes of product formation can be observed. At $k_La=25$ h^-1^, product formation begins more slowly and shown a clear exponential increase. In contrast, at $k_La=45$ h^-1^, production responds more quickly once oxygen limitation is alleviated. This behavior is due to the different oxygen availability and associated pellet state in the two cases.

Under lower $k_La$, the DOT drops to approximately 10% around *t*=50 h and remains at this low level for an extended period. At $k_La=45$ h^-1^, however, the minimum DOT remains around 20%, and the oxygen-limited phase is considerably shorter. Consequently, the time overlap between oxygen depletion and the onset of product formation varies with $k_La$, indicating a different effective contribution of glucose to secondary metabolite production under different shaking conditions. Additionally, the *α *-profiles shown in Figures (d) to (f) reveal an obvious increase in pellet activity with $k_La$, particularly in the profiles at *t = 48* h. This indicated an earlier transition from growth to product formation, despite similar overall productivity.

### Impacts of relative volume ratio $\gamma _V$

**Fig. 10:**
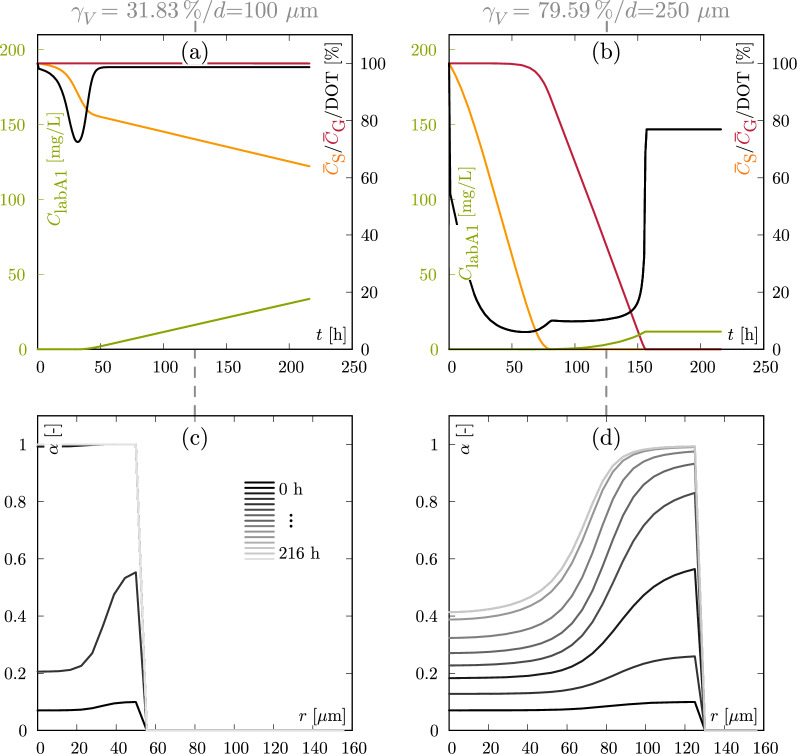
Simulation results of the pellet cultivation process under various hydrodynamic conditions with $k_La$ at: **a** and **d** 25 h^-1^, **b** and **e** 35 h^-1^, and **c** and **f** 45 h^-1^. The diagrams show the time-profiles of substrates and product, whereas the diagrams below illustrate the corresponding pellet states

The investigation into the influence of the impact of $\gamma _V$ essentially deals with how the biomass fraction and the associated mass transfer limitations affect overall productivity. As described in Sect. “Impact of volume partition on the cultivation process”, it is initially assumed that pellets with different diameters but the same nutrient content are simulated. The ensuing results are shown in Fig. 10.

It can be seen that product formation is reduced for both pellet diameters (100 *μ *m and 250 *μ *m). For pellets with a diameter of 100 *μ *m, the available nutrients are sufficient and there is no oxygen limitation. Consequently, the pellet reaches maturity within 48 h of cultivation, as shown in Fig. 10c. However, due to the limited biomass, the resulting productivity remains low. For pellets with a diameter of 250 *μ *m, the limitations in terms of nutrients and oxygen become apparent, requiring a longer cultivation time to achieve maturity, see (d). This is accompanied by a decline in productivity compared to the same cultivation time.

**Fig. 11:**
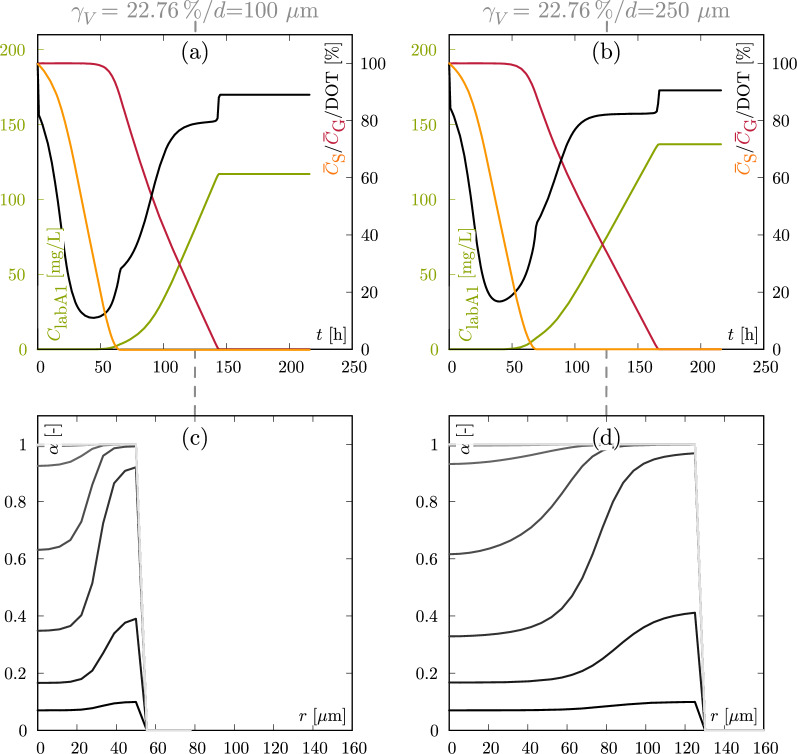
Simulation results of the pellet cultivation process with the same $\gamma _V = 22.76\%$: Glucose, glycerol, DOT, and labyrinthopeptin A1 concentrations at **a** *d = 100* *μ *m, **b** *d = 250* *μ *m. **c** and **d** show the corresponding pellet activity profiles shown, respectively

Under the second assumption mentioned in Sect. “Impact of volume partition on the cultivation process”, cultivations with the same relative volume ratio $\gamma _V$ of 22.76% are simulated. Therefore, the cultivation results and corresponding activity profiles of pellets with diameters (100 and 250 *μ *m) are shown in Fig. 11.

The product yield of pellets with a diameter of 100 *μ *m is lower than that of pellets with a diameter of 250 *μ *m. A comparison of the glucose profiles reveals no significant differences between the cases, however, a notable exception can be observed in glycerol consumption: Its depletion occurs earlier in the 100 *μ *m pellets than in the 250 *μ *m pellets. The lowest oxygen concentration was observed in the 100 *μ *m pellets, while the *α * profiles demonstrate a comparable pattern.

### Impacts of additional substrates

**Fig. 12:**
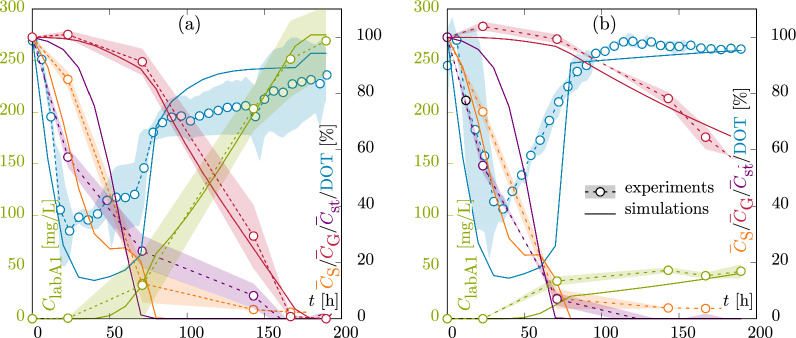
Comparison of simulation and experimental results of shake flask cultivation **a** salt-supplemented and **b** unsupplemented. Note, the simulation data are plotted as lines and the mean experimental measurements are shown as circles connected by dashed lines. The shaded area represented the standard deviation

To evaluate the model under different conditions, Fig. 12 shows the corresponding results of the extended model in Sect. “Extended model considering additional substrates”. This model incorporates additional starch in both salt-supplemented and unsupplemented cultivations, see (a) and (b). While the temporal profiles are generally similar to the results of this study, several kinetic parameters in the extended model must to be recalibrated to align with experimental observations.

In the case of salt-supplemented cultivation, the presence of starch as an additional carbon source doubles the total carbon supply compared to the pure glucose system in this study. Thus, the maximum glucose consumption rates ($k_\textrm{gS}$ and $k_\textrm{mS}$) increased to reflect the higher metabolic demand, resulting in a similar consumption time after 72 h of cultivation. Concomitantly, $k_La$ increased with the shaking frequency to prevent oxygen exhaustion in the shake flask, as since results with a double carbon source show a minimum DOT value of about 24% after 48 h of cultivation.

Furthermore, since the results show that the transition between glucose and glycerol occurs earlier, around 24 h, with the earlier production of labyrinthopeptin A1, the parameter governing $f^\textrm{SG}_\textrm{sw}$ was increased. Finally, $k_\alpha$ was reduced to align with the experimentally observed glycerol and DOT profiles. Figure 12a shows that, despite the difference in the lowest DOT due to the inadequate modeling of the hydrolysis mechanism, the recalibrated model parameters effectively capture the gradually increasing DOT slope between 45 and 65 h. The simulated DOT after 75 h exceeds the experimental results, which can be attributed to the relatively slow glucose and glycerol consumption observed in the experiment. However, the proposed kinetic model does not account for this long-tail effect. It should be emphasized that parameter optimization for glucose and starch considers only consumption duration and not nonlinear profiles over time. This is partly due to the limitations of the global modeling approach. Second, the rapid consumption of starch during the first 10 h of cultivation is not associated with an increase in glucose. This phenomenon cannot be adequately explained by hydrolysis mechanisms alone. This suggests that starch may be involved in more complex metabolic processes. To avoid overfitting and parameters that are physically unreasonable, results that better match glucose and glycerol profiles are not presented here because the corresponding $K_\textrm{st}$ and $k_\textrm{hyd}$ values were hundreds of times larger. Another source of error appears to be the consumption rate $q_\textrm{st}$, which is set too low at 1.11, see Eq. (28), and only considers the monomeric form of soluble starch.

For unsupplemented cultivation, reducing $k_\textrm{mG}$ decreases the consumption rate, leaving approximately 50% glycerol after 200 h of cultivation, see Fig. 12b. Consequently, the required oxygen is reduced, and the higher adjusted $k_La$ ensures that the DOT remains fully saturated after 72 h of cultivation rather than gradually recovering, as occurs in the salt-supplemented condition. Finally, despite the decrease in glycerol consumption, $\eta _\textrm{labA1}$ must be further reduced to account for the relatively low labyrinthopeptin A1 production. After reducing $k_\textrm{mG}$ by nearly fourfold to decrease the slopes of the DOT and glycerol profiles, a non-smooth turning point appears at 80 h is appears. This is due to the insufficient modeling of the hydrolysis mechanism and the non-long-tail effects in the simulated results. Despite the uncertainties in the first 72 h of cultivation, the determined $\eta _\textrm{labA1}$ of these two cases indicates that the salt-unsupplemented condition resulted in a reduction of approximately 50% in production efficiency. Furthermore, comparing the values of $k_\textrm{mG}$ and $k_\textrm{gG}$ reveals that the respiratory maintenance consumption rate of glycerol under the salt-unsupplemented condition slowed by approximately fourfold. This confirms that the salt-unsupplemented condition is unfavorable for pellet maintenance. Together, these two factors lead to a fivefold decrease in labyrinthopeptin A1 production.

## Discussion

### Culture performance and reproducibility

The *A. namibiensis* cultures exhibited diauxic growth. The amount of biomass increased significantly in the initial phase of glucose metabolism. This increase continued, albeit at a slower growth rate, in the subsequent phase of glycerol uptake. This growth behavior is consistent with the findings of previous studies [13, 20, 58]. The pH value remained stable throughout the cultivation process, but showed a slight shift to 7.5 in all four cultures analyzed during the last few days. This shift was probably caused either by a reduction in ammonium ion concentration, as reported by Tesche et al. [13], or by the production of secondary metabolite. For example, an increase in pH was observed during the production of the lantibiotic (NAI-107) in *Microbispora sp.*, which was associated with the utilization of amino acids and associated release of ammonia [59].

The maximum labyrinthopeptin A1 titer was reached on day 9 (216 h) of cultivation. Although previous studies documented slightly elevated concentrations, such as 273 mg/L [20], the minimal standard deviations observed in these quadruplicate experiments are remarkable compared to both standard filamentous cultures and previous studies with this actinomycete. In contrast to the earlier studies, a different cryoculture technique was used in this work. As stated by Tesche et al. [13] and Hanke et al.[58], the first pre-culture was inoculated with thawed agar pieces. However, this cryopreservation method is prone to variations between pre-cultures for two reasons: First, the method cannot guarantee homogeneous mycelial growth on agar plates. Second, the approach of producing solid cryostocks does not allow for homogenization or even aliquoting before freezing. In this experiment, a pellet culture was harvested during the early exponential growth phase. The culture was then thoroughly mixed by pipetting and evenly aliquoted before being frozen. Tesche et al. [13] reported higher mean total titers in cultures supplemented with 50 mM (NH_4_)_2_SO_4_, but significantly variation between replicates was also observed. These variations were attributed to significant foaming and heterogeneity of the pre-culture. Hanke et al. [58] also confirmed the finding of reduced reproducibility of cultures with the salt-supplementation. In the present study, inoculation of the pellets resulted in only minimal foaming, and the pellets remained relatively stable during the production phase (data not shown). There is a potential explanations for this observation: Dispersed mycelial fragments, which are more common when inoculating cultures with vegetative cells on agar pieces, could contribute to product formation. In a recent study, pellet cryocultures were used and lower reproducibility was observed in supplemented cultures compared to controls [20]. The significantly reduced standard deviations observed in Fig. 4 cannot therefore be explained solely by the different cryoinoculation method, but also by the mechanical homogenization of both the first and second pre-cultures. In summary, the low standard deviations observed here show a significant improvement in the reproducibility of the cultures, which was achieved through the combined effects of pellet-based cryopreservation and homogenization of the pre-cultures.

### Parameter analysis and comparison with conventional kinetic models

The experimental dataset comprises eight internally coupled dynamic curves covering substrate consumption, oxygen concentration at both the global and local domains, and product formation profiles. Since some curves are approximated by a polynomial representation, the number of inflection points and curvature changes indicates at least one characteristic degree of freedom per curve for the observed dynamics. With the exception of the predefined parameters and those derived directly from the existing literature, the model contains eight optimized parameters corresponding to the degrees of freedom implied by the experimental curves. Therefore, the parameter set is necessary and non-redundant and does not represent an overfitted flexible formulation.

The present study differs from studies that only considered oxygen or glucose consumption [24, 28, 29, 31, 40]. The model incorporates internal oxidation processes and couples the consumption of glucose and glycerol with oxygen. This enables a more integrated representation of the metabolic requirements within the pellet. As outlined in pioneering studies [24, 31, 40], the present model similarly takes into account both growth-associated and maintenance-related substrate consumption. The studies mentioned above postulated that the consumption rate is directly proportional to the biomass density. In contrast, the present work introduces the concept of $(c_\textrm{h} \cdot \alpha )$, serving as a surrogate for biomass. This term accumulates and evolves over cultivation, reflecting the underlying increase in biomass. Furthermore, the consumption rate is not determined solely by the equivalent pellet activity. The maintenance-related consumption is directly proportional to the pellet activity, meaning that mature pellets exhibit a higher maintenance requirement than newly formed pellets. However, the growth associated with consumption has a nonlinear relationship to pellet activity. As shown in Eq. (23), this nonlinear formulation captures two phenomena: Firstly, it captures the suppression of pellet growth under low-activity conditions. Secondly, it captures the reduced growth rate observed as the pellet approaches maturity.

Replacing biomass with the recently developed equivalent pellet activity means that the coefficients $k_{\textrm{m}i}$ and $k_{\textrm{g}i}$ in Eqs. (7), (8), (15) and (16) for maintenance and growth consumption are not comparable to those found in traditional biomass-dependent formulations. As shown in the study by Deffur et al. [31] on *A. niger*, the maintenance-related coefficient $k_\textrm{mO}$ and the growth-associated consumption coefficient $k_\textrm{gO}$ were defined as linear functions of biomass. When converted to the present formulation, the corresponding values are approximately 1.08 and 8.66 g/(Lh)^-1^, respectively. This comparison clearly indicates that $k_\textrm{gO}$ is higher than $k_\textrm{mO}$, which is consistent with the behavior predicted by this model.

Although no direct measurements of pellet activity are available, the maximum growth rate obtained in this study cannot be directly compared with values reported in other studies. Reported maximum growth rates for *A. niger* range from 0.06 to 0.30 h^-1^ [29, 31, 40, 60]. These rates quantify the increase in pellet biomass and therefore represent a fundamentally different process. In these studies, pellet growth is governed by time and Monod-type dependence on available substrate concentration.

In contrast, the present model defines the maximum growth rate as the outcome of evolving pellet activity rather than biomass accumulation alone. Pellet growth depends on time and substrate availability but is also coupled to the consumption rate of the cultivation substrate. This coupling provides a more detailed representation of energy transfer from nutrient uptake to pellet growth and product formation.

### Limitations of this work

Despite the results presented here, there are still some limitations in this work. First, oxygen supply is assumed to be constant in the fluid domain. In reality, however, the oxygen concentration varies over time during the cultivation, while the viscosity of the culture changes dynamically with substrate consumption and pellet growth. Furthermore, the fluid-air interface fluctuates continuously due to the orbital shaking at 180 rpm. These factors mean that the actual oxygen transfer conditions are highly dynamic, and oxygen transfer depends on transient concentration gradients rather than uniform supply. Therefore, the applied $k_La$ value cannot fully capture the transient oxygen transfer behavior occurring in the shake flask cultivation. Second, the development of *α * is largely based on assumptions that cannot be quantitatively validated. The initial pellet state $\alpha _0$ is set to 0.1 without a physical unit, and it is assumed that pellet state occurs at *α =0*, as shown in Table 2. The *α * value acts as internal microbial state variables that reflect the pellet growth state, which is a model assumption rather than a directly measurable physical quantity. When applying this model to other microbial cultivations, the *α * formulation may not be directly transferable, and its dynamics and initial value may differ substantially depending on the strains, pre-culture and cultivation conditions.

Additionally, another aspect of the cultivation behavior deserves attention. Labyrinthopeptin A1 continued to accumulate for several hours after both carbon sources were depleted. This behavior is consistent with the characteristics of non-growth-associated secondary metabolites, whose formation is not directly coupled to biomass growth or the presence of extracellular substrates. Even after external carbon sources are depleted, cells may have sufficient intracellular precursor pools and metabolic activity to sustain secondary metabolism for a period of time, thereby uncoupling the production of labyrinthopeptin A1 from the availability of extracellular carbon. Additionally, the synthesized product may be retained within the hyphal network and released gradually, lacking a sufficient spatial gradient for mass transfer in the porous structure.

To consider the time-delay effect in future work, Eq. (24) can be extended by introducing an explicit intracellular reserve pool. For example, the variable *α * is an accumulated transformation of carbon source, see Eq. (23), and its catabolism can induce further product formation. Since this mechanism is expected to be relevant only under carbon-limited conditions, an additional activation function is required to suppress this term’s contribution during the early stages of cultivation. Furthermore, determining both the functional forms and the associated parameters requires more careful and dedicated experimental design. For example, experiments with specially modified initial and boundary conditions should be conducted to detect dependence on *α * and define the activation criterion. This can be regarded as an extension of the framework shown in Fig. 1.

Additionally, the effect of hydrodynamic loading conditions can be described by introducing evolution equations that describe temporal changes in morphological parameters. These parameters can serve as additional variables in the coupled load-morphology-kinetics model. These changes will subsequently influence the mass transfer and growth kinetics of the pellet. However, developing and calibrating these evolution equations requires more comprehensive experimental characterization and numerical analysis. For instance, numerical simulations could quantify fluid-solid interactions under different shaking conditions. In parallel, time-resolved oxygen profiling of pellets of various sizes would help establish the relationship between morphological evolution and mass transfer. To achieve a more accurate parameterization, future work could utilize a microfluidic setup to track the growth of individual pellets over time.

Furthermore, this framework can be extended to include an upscaling study, although direct upscaling from lab to pilot scale is challenging. For example, under the heterogeneous conditions of a stirred tank reactor, the modeling approach based on volume-averaged representative pellet and its associated domains must be modified. This requires morphological statistics on the pellets and experimental characterization of heterogeneous concentration fields. Besides the different boundary conditions at the pilot scale, the pellet-related mechanisms in the model must be reconsidered since they respond to the surrounding environment. While the basic modeling framework and the oxygen-substrate stoichiometry derived from the oxidation process remain valid, the critical parameters of the Monod functions require recalibration. This is because the pellets in a stirred tank reactor are exposed to process disturbances that drive pellet behavior far beyond the gentle fluctuations observed at lab scale. Identifying these parameters requires experiments under multiple process conditions and the integration of numerical tools that quantitatively embed unmeasurable effects, such as heterogenous flow fields and fluid-solid interactions, into the parameterization.

## Conclusions

This study presents a mechanistic framework linking substrate consumption, oxygen limitation, pellet state evolution, and labyrinthopeptin A1 production during *A. namibiensis* cultivation. By formulating the substrate uptake rate as a function of equivalent pellet activity, the model captures the pellet’s heterogeneous metabolic states during cultivation. Assuming complete oxidation, the oxidation reaction couples oxygen consumption with consumption of carbon substrates (glucose and glycerol) via fixed stoichiometric coefficients. The framework incorporates the diauxic shift observed during growth on multiple carbon sources, enabling the model to represent the sequential substrate utilization mechanism. The model also distinguishes between growth-associated and maintenance metabolism, partioning carbon source consumption into biomass formation and non-growth energy requirements. As the pellet matures, a portion of the available carbon sources is redirected toward maintenance demands and labyrinthopeptin A1 biosynthesis. This separation is essential for secondary metabolites. Nevertheless, by accounting for both the pellet and the surrounding fluid domains, the model integrates reaction kinetics with mass-transfer limitations and pellet state evolution. This integrated description provides a foundation for understanding the pellet growth kinetics and the formation of secondary metabolites.

## Data Availability

The data collected as part of this study are available upon request.
